# Draft Genome Sequence of Empedobacter (Formerly Wautersiella) falsenii comb. nov. Wf282, a Strain Isolated from a Cervical Neck Abscess

**DOI:** 10.1128/genomeA.00235-15

**Published:** 2015-04-02

**Authors:** German Matías Traglia, Chelsea Dixon, Kevin Chiem, Marisa Almuzara, Claudia Barberis, Sabrina Montaña, Cindy Merino, María Alejandra Mussi, Marcelo E. Tolmasky, Andres Iriarte, Carlos Vay, María Soledad Ramírez

**Affiliations:** a Instituto de Microbiología y Parasitología Médica (IMPaM, UBA-CONICET), Buenos Aires, Argentina; b Laboratorio de Bacteriología Clínica, Departamento de Bioquímica Clínica, Hospital de Clínicas José de San Martín, Facultad de Farmacia y Bioquímica, Buenos Aires, Argentina; c Departamentos de Bioquímica y Genómica Microbianas and Genómica, IIBCE, Montevideo, Uruguay; d Departamento de Desarrollo Biotecnológico, Facultad de Medicina, Instituto de Higiene, UdelaR, Montevideo, Uruguay; e Centro de Estudios Fotosintéticos y Bioquímicos (CEFOBI- CONICET), Rosario, Argentina; f Department of Biological Science, Center for Applied Biotechnology Studies, California State University Fullerton, Fullerton, California, USA

## Abstract

Empedobacter (formerly Wautersiella) falsenii comb. nov. strain Wf282 was isolated from a cervical neck abscess sample from an 18-year-old female patient. The isolate was resistant to many antibiotics, including meropenem and colistin. The total DNA from the multidrug-resistant E. falsenii comb. nov. Wf282 clinical isolate was sequenced.

## GENOME ANNOUNCEMENT

Empedobacter (formerly Wautersiella) falsenii comb. nov. was first described in 2006 and renamed in 2014 (1, 2). Its phenotype resembles members of the genera Chryseobacterium and Empedobacter. E. falsenii comb. nov. is a nonmotile Gram-negative rod that grows aerobically at 20, 30, and 37°C on standard medium, such as tryptic soy agar or blood agar. In the year 2012, the first isolation of Wautersiella falsenii from a urine sample of an infant with a complicated urinary tract infection was described (3).

E. falsenii comb. nov. strain Wf282 was isolated from a cervical neck abscess sample from an 18-year-old female patient who was admitted to the otolaryngology service with acute otitis media. The nonfermenting Gram-negative rods were identified as E. falsenii using a matrix-assisted laser desorption ionization–time of flight mass spectrometer (MALDI-TOF MS) (Bruker, Germany), with a score of 2.074 (probable species-level identification). Antibiotic susceptibility testing was performed using the Vitek 2 system, employing the panel AST-079 (Gram-negative susceptibility [GNS] card). The MIC results were interpreted using the CLSI breakpoints (4) for other non-*Enterobacteriaceae*, except for ampicillin, cephalothin, and cefoxitin, for which those for *Enterobacteriaceae* were used. E. falsenii comb. nov. strain Wf282 was resistant to ampicillin (≥32 µg/ml), ampicillin-sulbactam (≥32 µg/ml), cephalothin (≥32 µg/ml), meropenem (≥16 µg/ml), and colistin (8 µg/ml); intermediate to piperacillin-tazobactam (64 µg/ml), cefotaxime (16 µg/ml), ceftazidime (16 µg/ml), imipenem (8 µg/ml), and ciprofloxacin (2 µg/ml); and susceptible to cefepime (4 µg/ml), amikacin (8 µg/ml), gentamicin (2 µg/ml), and trimethoprim-sulfamethoxazole (2 µg/ml).

Whole-genome shotgun sequencing was performed using Illumina MiSeq-I, using Nextera XT libraries for sample preparation. The reads were assembled with the Ray assembler (http://denovoassembler.sourceforge.net). A total of 1,076,192 high-quality paired-end reads were produced, with an average insertion size of 825 bp. *De novo* assembly was performed with the SPAdes assembler version 3.1.0 (5), using a preassembly approach with Velvet (6). Of the generated reads, 99.9% were mapped, resulting in a mean nucleotide coverage of 65× (and a *k*-mer coverage of 29.9). The corrected reads showed an average length of 230 bp. The assembled contigs sum 3,738,626 base pairs, with an *N*_50_ contig size of 113,944 (max length, 279,129) and a G+C content of 32.24%.

Since this isolate is resistant to many antibiotics, including meropenem and colistin, the future detailed analysis of the genome of this strain (Wf282) might lead us to find novel antibiotic resistance traits that may explain its phenotype, as well as its potential role as a reservoir of antibiotic resistance genes.

### Nucleotide sequence accession numbers.

This whole-genome shotgun project has been deposited at DDBJ/EMBL/GenBank under the accession no. JSYQ00000000. The version described in this paper is version JSYQ01000000.
